# Proteomic Dissection of Exosome Cargo: Progress and Future Perspectives

**DOI:** 10.3390/cancers15174292

**Published:** 2023-08-28

**Authors:** Shahab Uddin, Aamir Ahmad

**Affiliations:** 1 Translational Research Institute, Academic Health System, Hamad Medical Corporation, Doha 3050, Qatar; skhan34@hamad.qa; 2 Dermatology Institute, Academic Health System, Hamad Medical Corporation, Doha 3050, Qatar; 3 Laboratory Animal Research Center, Qatar University, Doha 2713, Qatar; 4 Department of Biosciences, Integral University, Lucknow 226026, UP, India; 5 Department of Dermatology and Venereology, Rumailah Hospital, Hamad Medical Corporation, Doha 3050, Qatar; 6 Department of Bioengineering, Integral University, Lucknow 226026, UP, India

The interest in exosomes in cancer research and treatment has increased exponentially in the past few years. Exosomes are small extracellular vesicles released from all cell types which function as cargo carriers, thus facilitating cell-to-cell signaling by delivering the cargo to recipient cells [1]. The cargo carried by typical exosomes can be very diverse, ranging from proteins to nucleic acids to metabolites and lipids. For tumor cells, exosomes are believed to confer survival advantages by modulating immune response and affecting angiogenesis, metastasis and resistance against therapies [2]. These biological functions ascribed to exosomes are possible all because of their cargo contents [3,4]. As the interest in exosomes has intensified, so has the scrutiny of their contents. The review article ‘Proteomic Analysis of Exosomes for Discovery of Protein Biomarkers for Prostate and Bladder Cancer’ by Wang et al. [5], published as part of Special Issue ‘The Cancer Proteome’, focused on the analysis of one specific cargo constituent of exosomes. The specific cargo constituent was proteins, and the analysis was proteomics-based. The idea that there are differences in proteome contents of ‘normal’ vs. ‘tumor’ cells is well accepted. For more than two decades, the idea of utilizing proteomics-based analyses to study these differences has been circulated [6,7]. Fast forward to the present-day, and proteomics-based cancer biomarker discovery research still remains a hot topic [8], with specific attention paid to the proteomic analyses of extravesicular vesicles, including exosomes [9].

The review article by Wang et al. [5] focuses on two specific and common urologic cancers, prostate and bladder cancers. Before discussing the state of knowledge on the proteome content of exosomes from prostate and bladder cancers, the authors first discuss the various exosome isolation and characterization methodologies. This is critical because sample preparation methods, in this case, exosome isolation, can impact the downstream proteomics-based detection and analyses. One needs to keep in mind that thousands to tens of thousands of proteins can potentially be listed in proteomic analyses of exosomes [5,10,11]. Add to this the challenge of (a) contamination in biofluids with proteins that are not from exosomes and (b) the overlapping size and morphology of exosomes with microvesicles (exosomes and microvesicles can often have very distinct cargo and associated functions), and suddenly the focus on well defined and validated isolation methods for exosomes starts making sense. The six exosome-isolation methods that the authors discuss in this review article are ultracentrifugation, density gradient centrifugation, size exclusion chromatography, ultrafiltration, affinity isolation and precipitation. The advantages, as well as the disadvantages/limitations, of these isolation methods are listed. Following isolation, the next important task is the characterization of exosomes to make sure of their purity. A number of techniques are available for this task, and they include dynamic light scattering and nanoparticle tracking analysis for assessing the size and distribution of exosomes; transmission electron microscopy for structural details; cryo-electron microscopy for the morphology of exosomes; flow cytometry for qualitative as well as quantitative analysis and Western blotting and ELISA for the determination of exosome purity [5]. Once the exosomes-preparations are deemed pure, proteomic analysis mostly consists of mass spectrometry (MS)-based methodology.

Following the discussion on exosome isolation and characterization, the article [5] shifts its focus to the actual protein biomarkers discoveries in prostate and bladder cancers, as revealed by proteomic analyses on exosomes from these cancers. The authors start with diagnosis and make a case for the need to discover novel diagnostic biomarkers. For prostate cancers, authors argue that PSA (prostate-specific antigen), the commonly used prostate cancer diagnostic marker, is probably not reliable as it is particularly unable to differentially diagnose benign vs. advanced disease and is a major cause of overdiagnosis and unnecessary overtreatment [5,12,13]. For bladder cancers, they make a case that the highly specific cytology test has low sensitivity [14], whereas the gold standard cystoscopy is invasive and comes with the risk of developing urinary infections [15]. Thus, the authors make a case for the need to discover novel diagnostic biomarkers for both prostate and bladder cancers. Authors then summarize the reports on MS-based proteomic analyses of exosomes from urine, plasma/serum and cell culture media for prostate cancer and the exosomes from urine and cell lines for bladder cancer. Clearly, the cell lines-based studies are relatively easy to perform, and, in the patients-based studies, urine is a more relevant biofluid in addition to sample collection being non-invasive when compared to plasma/serum, and therefore more widely studied. It is revealed that ultracentrifuge-based exosome-isolation, in combination with density gradient centrifugation, is the most preferred method for downstream proteomic analysis because of the highly pure exosomes quality, even though the overall method can be very lengthy and impractical for large cohorts. 

A detailed discussion of different protein biomarkers revealed, in several individual studies on prostate cancer listed, a few key proteins that could be potential prostate cancer biomarkers. These include FABP5 (fatty acid-binding protein 5), TM256, LAMTOR1, VATL and ADIRF [5]. However, the authors make a point that very low overlap was observed among the published reports in terms of protein biomarkers, and this can be attributed to inherent differences in the study designs, low sample sizes, urine vs. cell lines-based analyses, etc. When a more direct comparison was carried out between individual studies, FABP5 and LAMTOR proteins did stand out, with some overlap and detection across studies. The conclusions were similar for bladder cancer exosome proteosome analyses, and almost no overlapping proteins were identified across the studies. All the studies discussed in the article listed unique proteins, such as TMPRSS2, TPP1, FOLR1, RALB, RAB35, SLC4A1, TACSTD2 and urothelial carcinoma-associated proteins. This was again attributed to utterly different study designs and sample sizes. The article even listed two studies in prostate cancer that focused on proteomic analysis of exosomes in light of racial cancer disparities. The racial disparities, i.e., the differences between individual races in cancer incidence and mortality, particularly between Caucasians (European Americans) and African Americans, have been of great interest in the United States [16,17]. Again, no consensus in protein biomarkers could be concluded, even though Filamin A stood out in one of the studies as an African American-specific protein. One of the drawbacks identified in these studies was the origin of exosomes as these were plasma/serum-based studies, and apparently, the samples were not depleted of high-abundance proteins. Compared to the patient-derived exosomes discussed so far, the exosomes from cell culture models are supposed to be more consistent in terms of their protein cargo, primarily because all the cells in a culture are of the exact same type. However, because of cell-line-specific changes, the results are still not comparable when proteomic analysis of exosomes from one cell line is compared to that from another, even though they may ‘represent’ the same cancer.

Based on the discussion in this review article by Wang et al. [5], it is apparent that despite realizing the promise of proteomic analysis of exosomal cargo in cancer diagnosis and progression over the last two decades, the overall progress is minimal. Perhaps, the initial step, the isolation of exosomes, deserves the blame. The overall field of proteomics has witnessed a lot of progress regarding state-of-the-art quantitative proteomic platforms. However, there is still a lack of highly effective, reliable and robust methods for rapid exosome isolation, particularly when there is a large number of samples [5]. Additionally, since biofluids such as plasma/serum are limited in terms of quantities that can be obtained from patients, future exosome isolation techniques also need to ensure high yields of exosomes. Once a more reproducible and much more standardized exosome isolation methodology is in place, identification and validation of novel protein biomarkers from exosome cargo will inevitably follow.
